# Sevoflurane Preconditioning Alleviates Posttraumatic Stress Disorder—Induced Apoptosis in the Hippocampus via the EZH2-Regulated Akt/mTOR Axis and Improves Synaptic Plasticity

**DOI:** 10.1007/s12031-023-02114-1

**Published:** 2023-03-17

**Authors:** Tingting Gu, Chang Xu, Xiaozhou Meng, Dapeng Gao, Guanghao Jiang, Anqi Yin, Qingzhen Liu, Lidong Zhang

**Affiliations:** 1 Department of Anesthesiology, Jinling Hospital, Medical School of Nanjing University, Nanjing, China; 2 Department of Anesthesiology, Jinling Hospital, Medical College of Nanjing Medical University, Nanjing, China; 3 Department of Anesthesiology, Yancheng First Hospital, Affiliated Hospital of Nanjing University Medical School, Yancheng, China

**Keywords:** Sevoflurane, EZH2, PTSD, Apoptosis

## Abstract

Posttraumatic stress disorder (PTSD) is a persistent and severe psychological and mental disorder resulting from experiences of serious trauma or stress and is suffered by many individuals. Previous studies have shown that pretreatment with sevoflurane is efficient in reducing the incidence of PTSD. However, we require a more comprehensive understanding of the specific mechanisms by which sevoflurane works. Enhancer of zeste homolog 2 (EZH2) has been reported to be regulated by sevoflurane, and to improve patient cognition. In this study, we aimed to explore the mechanisms of sevoflurane and the role of EZH2 in PTSD cases. We explored the effects of sevoflurane and EPZ-6438 (inhibitor of EZH2) on rat behavior, followed by an investigation of EZH2 mRNA and protein expression. The effects of sevoflurane and EZH2 on neuronal survival were assessed by western blotting and TUNEL staining, while western blotting was used to examine the expression of PSD95 and the AKT/mTOR proteins. Sevoflurane preconditioning restored EZH2 expression and significantly inhibited apoptosis by regulating phosphorylation of the AKT/mTOR pathway. Synaptic plasticity was also significantly improved. These results suggest that pretreatment with sevoflurane could play an important role in PTSD prevention by regulating EZH2 expression.

**Supplementary Information:**

The online version contains supplementary material available at 10.1007/s12031-023-02114-1.

## Introduction

Posttraumatic stress disorder (PTSD) is a psychological disorder that develops after exposure to a traumatic event in which grave physical harm occurred or was threatened (Bomyea et al. 2012). Long-term anxiety and recurring feelings of fear are characteristic of PTSD, causing distress to patients, and impacting their daily lives and long-term social behavior. Unfortunately, effective means of preventing and treating PTSD are limited and it is thus critical to explore more effective therapeutic strategies.

Sevoflurane is widely used in clinical practice as an inhaled anesthetic. It induces rapid unconsciousness in patients, allows immediate awakening after surgery, is highly effective as an anesthetic, and has fewer obvious side effects compared with other types of anesthetics (Fang et al. 2022). In recent years, preconditioning/postconditioning with sevoflurane has been discovered to be effective against various forms of cognitive impairment and the protective effects of sevoflurane in cognition and behavior have attracted much attention. It has been found that preconditioning with sevoflurane can significantly alleviate PTSD symptoms and reduce apoptosis by regulating brain-derived neurotrophic factor (BDNF) expression and phosphorylation of the protein kinase B (Akt)/glycogen synthase kinase-3β (GSK-3β) signaling pathway (Chen et al. 2015). Administration of sevoflurane was also found to improve apoptosis in traumatic brain injury (TBI) via the (phosphatidylinositol-3-kinase) PI3K/AKT signaling pathway (He et al. 2018). However, the potential mechanisms involved in the neuroprotective functions of sevoflurane require more exploration.

A balance between methylation and demethylation is central to maintaining physical and mental health. A growing body of research suggests that there is a direct relationship between methylation disorders and cognitive dysfunction (Wheater et al. 2020). Methylation has profound effects on many aspects of the nervous system, including neurotransmitter production, mood regulation, motor function, and cognitive performance. Enhancer of zeste homolog 2 (EZH2), known to modulate cancer development, is expressed strongly in the hippocampus. EZH2 can combine directly with S-adenosylmethionine (SAM), transferring a methyl group to the histone H3K27 position to form H3K27me3, which can suppress downstream gene expression. EZH2 has been found to play an important role in apoptosis, autophagy, and cell cycle progression (Duan et al. 2020). Previous studies have demonstrated that EZH2 expression affects the phosphorylation of AKT/mechanistic target of rapamycin (mTOR) via the regulation of H3K27me3 and repression of PTEN, suggesting a novel epigenetic mechanism in the process of memory reconsolidation. Therefore, regulation of EZH2 may be an important therapeutic strategy for attenuating mal-memories as reconsolidation principles (Jarome et al. 2018). Moreover, many studies have demonstrated that sevoflurane can regulate EZH2 expression and exert neuroprotective effects (Wang et al. 2022; Xue et al. 2019). Therefore, we speculated that reduced EZH2 expression in PTSD may promote apoptosis. Sevoflurane treatment may effectively restore EZH2 expression in PTSD models with subsequent neuroprotective effects. In this study, we explored the changes in EZH2 expression in PTSD cases and whether sevoflurane can improve PTSD symptoms via its regulation of EZH2. We also researched downstream pathways to discover the epigenetic mechanisms affected by EZH2 regulation, and we measured changes in synaptic plasticity to further evaluate the effects of sevoflurane and EZH2 in PTSD.

## Materials and Methods

### Experimental Design

Rats were randomly allocated to different experimental groups. For experiment A, rats were randomly divided into the control and PTSD groups. Rats in the PTSD group were subjected to inescapable foot shock (IFS) procedures, and control rats were placed in the same chamber without experiencing any shocks. Then, all rats were sacrificed for the determination of EZH2 expression. For experiment B, rats were randomly divided into five groups: control (C), PTSD (P), PTSD + sevoflurane (PS), PTSD + sevoflurane + DMSO (PSD), and PTSD + sevoflurane + EPZ-6438 (PSE). Sevoflurane pretreatment was given 30 min before the IFS procedure. Rats in the PS, PSD, and PSE groups received 2.4% sevoflurane (Baxter, USA) + 98% O_2_ in a sealed chamber for 1 h (Chen et al. 2015; Wang et al. 2022). EPZ-6438 (also known as Tazemetostat, TargetMol, USA) (Zhang et al. 2015) was dissolved in dimethyl sulfoxide (DMSO) to a stock solution of 5 mg/ml and then diluted in saline to a working concentration of 0.1 mg/ml. After sevoflurane treatment, the rats in the PSE group were injected intraperitoneally with EPZ-6438 at a dosage of 3 mg/kg/day, which has been shown to maintain its effect for up to 3 weeks (Luo et al. 2020). To eliminate systemic error, rats in the PSD group were given 2% DMSO as a vehicle via intraperitoneal injection. For experiment C, rats were randomly divided into five groups: control (C), PTSD (P), PTSD + sevoflurane (PS), PTSD + mTOR inhibitor-3 (PI), and PTSD + sevoflurane + mTOR inhibitor-3 (PSI). Rats in the PI and PSI groups were injected with mTOR inhibitor-3 (MCE, USA) dissolved in DMSO and saline.

### Microarray Data

To investigate differences in *Ezh2* gene expression between PTSD and healthy rats, the microarray dataset GSE860 was downloaded from the Gene Expression Omnibus (GEO) database. Data from four months after trauma (with complete research results) were used in this study. Rats with reduced symptoms were selected for the control group (GSM13136, GSM13137, GSM13151, GSM13153, GSM13154), while those that showed no obvious decrease in symptoms were chosen for the PTSD group (GSM12955, GSM13122, GSM13124, GSM13127, GSM13130). Differences in the expression of *Ezh2* were identified by GEO2R (a commonly used online tool that can compare two or more datasets in a GEO series to identify differentially expressed genes across experimental conditions).

### Animals

Sprague–Dawley (SD) rats (6–8 weeks old, weighing 180–220 g) were purchased from Vital River Experimental Animal Cooperation (Nanjing, China). Rats were kept in clean, tidy, standard cages (six rats per cage) and maintained at 22 °C, a relative humidity of 60%, and a 12-h light–dark cycle. Rats were allowed an adaptation period of 1 week with free access to food and water before the experiments.

### IFS Procedure

IFS is an effective approach for the reproduction of symptoms such as avoidance, stress, and persistence of fear memory. Long-lasting behavioral and physiological effects can be maintained for up to 3 weeks and are even aggravated over time. In this study, rats were placed in a shock chamber with an electrified grid floor (30 × 26 × 22 cm) for 5 min, after which they received a sound stimulus at 5 kHz and 75 dB (conditioned stimulus) for 30 s. Following the stimulus, 10 shocks (2 mA) of 2 s in duration were given randomly over 15 min (Ji et al. 2014; Sun et al. 2016). Finally, the rats were kept in the chamber for another 60 s before they were returned to their home cages. Control rats were placed in the same chamber for an equal amount of time and subjected to the same sound stimulations while receiving no foot shocks. The shock chamber was cleaned with 75% ethanol before each session.

### Behavioral Experiments

Behavioral tests were conducted in a quiet, dark room during the light cycle (08:00–16:00) by an experimenter who was blinded to the animal grouping. The tests were conducted in the following order: open-field test (OFT), elevated plus maze test (EPM), and fear conditioning test (FC).

#### OFT

The OFT device consisted of a dark box (95 × 95 × 95 cm) divided equally into 25 small squares at the bottom. Before the tests, the rats were kept in the behavior room to adapt to the new environment for 1 h. During the test, rats were placed alone in the central area and allowed to freely explore the field for 5 min. Their activity was recorded with a camera placed above the apparatus, and the behavior of each rat was recorded and analyzed with a video-tracking system. After each session, the surface of the apparatus was cleaned with 75% ethanol. The time spent in the central area was recorded and analyzed.

#### EPM

EPM is a well-known procedure used to test anxiety-like behavior in animals. A standard EPM with two closed arms (50 × 10 × 40 cm), two open arms (50 × 10 cm), and a central area (10 × 10 cm) were placed at a height of 90 cm from the ground. To acclimatize the animals to the laboratory conditions, they were moved to the behavior room 1 h before the experiment. Rats were placed individually in the central area, facing an open arm; then, they were allowed to explore freely for 5 min. The time spent in the open arms was recorded for the evaluation of anxiety. The apparatus was cleaned with 75% ethanol after each session.

#### FC

After adaptation to the behavior room for 1 h, rats were placed in the same shock chamber used in the IFS procedure, which was equipped with an electrified grid floor (contextual reminder). They were allowed to explore freely for 5 min without any electric shocks or sound stimuli. After a rest for 2 h, they were put in another chamber with a different appearance and smell for 5 min, followed by 30 s of the sound reminder (the same condition as IFS procedure). During the tests, rat freezing time was recorded. Freezing time caused by exposure to the shock chamber and the sound reminder was recorded as a measurement of contextual fear and conditional fear, respectively. The chamber was cleaned with 75% ethanol after each session.

### Immunofluorescence

Rats were anesthetized and transcardially perfused with phosphate-buffered saline (PBS) followed by 4% paraformaldehyde. The brains were quickly extracted and fixed in paraformaldehyde at 4 °C before immersion in 30% sucrose overnight for dehydration. The brain tissues were cut into 30-mm-thick frozen sections, which were blocked with 1% BSA for 1 h at room temperature and incubated with the primary rabbit anti-EZH2 monoclonal antibody (CST, 5246; 1:200) at a 1:200 dilution at 4 °C. The sections were then washed three times with TBST and incubated with the secondary antibody (Servicebio, GB23303, 1:2000) for 1 h at room temperature. After three further washes in PBS, the nuclei were stained with 4,6-diamidino-2-pheny-lindole (DAPI; Life Technologies). Fluorescent images were recorded with a fluorescence microscope (Olympus, Japan).

### TUNEL

To calculate the rate of apoptosis in the hippocampal cells of the rats, TUNEL assays were used to detect DNA fragmentation in the nuclei. The rats were anesthetized and transcardially perfused with PBS and 4% paraformaldehyde, after which the entire brain was removed and stored in 4% paraformaldehyde at 4 °C. The brains were washed three times with running water, dehydrated in an alcohol sequence of 70%, 80%, 90%, 95%, and 100% for 30 min, respectively, and embedded in paraffin. The samples were cut into 4-μm sections, which were incubated in PBS containing 0.1% Triton X-100 and 0.1% sodium citrate for 8 min, then washed three times. The sections were washed in 0.3% H_2_O_2_ in methanol for 10 min and incubated with the reagent from a TUNEL cell apoptosis kit (C1086, Beyotime, China) for 1 h at 37 °C. After imaging under an inverted fluorescence microscope (HB050, Germany), the numbers of apoptotic cells in the hippocampi were calculated in six serial sections using Image-Pro Plus software.

### Western blotting

After the behavioral tests, the rats were anesthetized and transcardially perfused with PBS. The brains were removed and dissected on ice to collect the hippocampi. The hippocampi were then stored individually in cryotubes at −80 °C. The samples were lysed in RIPA buffer (Beyotime, P0013B) containing PMSF (Servicebio, G2008) and phosphatase inhibitor (Beyotime, P1081), followed by thorough mixing and centrifugation (12,000 rpm, 8 min). The protein concentrations in the supernatants were measured with a BCA Protein Assay Kit (Beyotime, P0010). SDS loading buffer was then added to each sample before vortexing and boiling for 5 min. Samples (10 μg per lane) were separated on 10% sodium dodecyl sulfate–polyacrylamide gel electrophoresis and transferred to polyvinylidene difluoride membranes. Following blocking with non-fat milk, the membranes were incubated with the following primary antibodies: anti-EZH2 (CST, 5246; 1:1000), anti-AKT monoclonal antibody (Proteintech, 60,302–2-lg, 1:5000), anti-p-AKT (CST, 9271, 1:1000), anti-mTOR (CST, 2983, 1:1000), anti-p-mTOR (CST, 5536, 1:1000), anti-BAX polyclonal antibody (Proteintech, 50,599–2-lg, 1:1000), anti-Bcl2 monoclonal antibody (Proteintech, 68,103–2-lg, 1:1000), anti-PSD95 (CST, 3409, 1:1000), polyclonal rabbit anti-GAPDH (Proteintech, 10,494–1-AP, 1:5000), and anti-β-actin (Servicebio, GB12001, 1:5000) overnight at 4 °C. Then, the membranes were incubated with secondary antibodies (Servicebio, GB23303, 1:5000; Proteintech, HRP-60008, 1:5000) for 1 h. The bands were visualized using an ECL detection kit, and the band intensities were measured with Image J software.

### Statistical analysis

Data were analyzed with the SPSS Statistics 22.0 and GraphPad Prism 9 software. All data are expressed as mean ± standard error of the mean (SEM). Differences between groups were all analyzed with one-way ANOVA and Tukey’s multiple comparison test. A difference between groups was considered significant when the *p*-value was < 0.05.

## Results

### Expression of EZH2 Is Downregulated in PTSD Rats

**Fig. 1:**
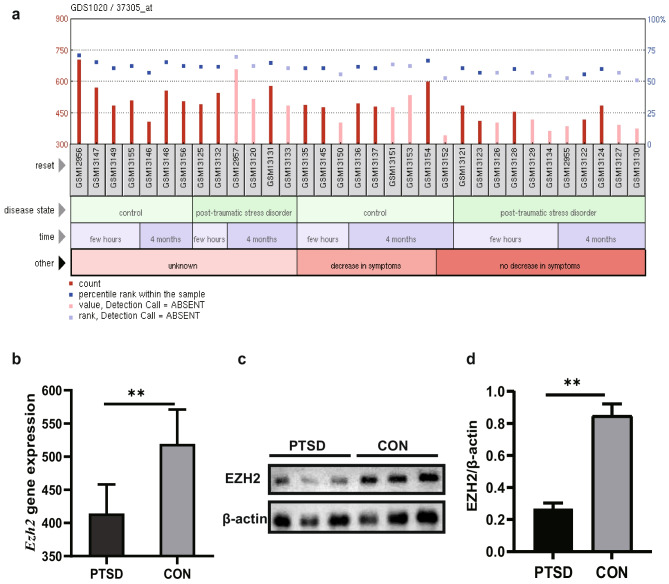
Expression of EZH2 is downregulated in PTSD rats. **a** Data from GEO: levels of *Ezh2* gene expression in each case (each GSM number represents a case). **b** Bar graph shows lower levels of *Ezh2* gene expression in the PTSD group than in the control group. **c** Western blot showing EZH2 protein expression. **d** The density ratio of EZH2/β-actin in the PTSD group was significantly lower than in the control group. *p < 0.05,**p < 0.1. All data are presented as means ± SEM; data analyzed via one-way analysis of variance

**Fig. 2:**
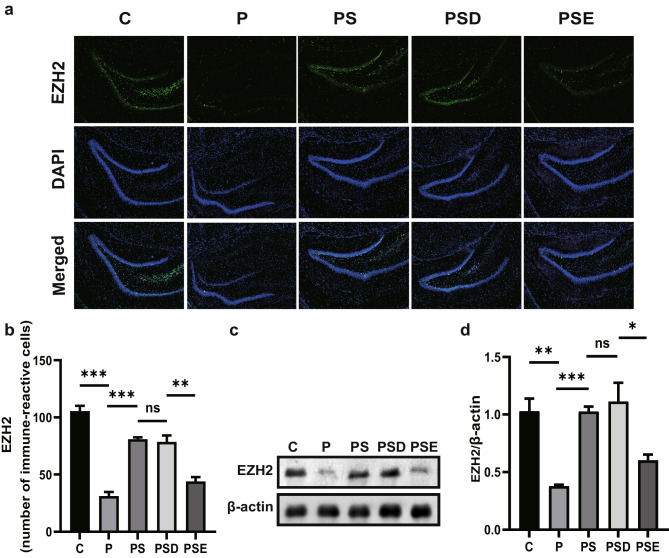
Pretreatment with sevoflurane regulates EZH2 expression in rat hippocampus. Expression of EZH2 in the five groups. **a**, **c** Effects of sevoflurane treatment and EZH2 inhibitors on EZH2 expression, shown by western blotting and immunofluorescence. **b** Bar graph showing quantitative measurements of immunoreactive cells. **c** Bar graph showing EZH2 protein expression. *p < 0.05, **p < 0.1, ***p < 0.01, ****p < 0.001; ns indicates no significant difference. All data are shown as means ± SEM; data were analyzed via one-way analysis of variance

To explore differences in *Ezh2* gene expression between PTSD and normal rats, we analyzed a dataset downloaded from the GEO. Rats showing an obvious decrease in symptoms were allocated to the control group, while those that did not display improvements in symptoms were placed in the PTSD group (Fig. 1a). *Ezh2* expression was markedly lower in the PTSD group (Fig. 1b). To verify changes in the EZH2 protein expression, we further conducted western blotting and immunofluorescence analyses of rats’ brains from the different groups (Figs. 1c and 2a). The analysis (Fig. 1d) showed a clear difference in EZH2 protein expression between the two groups. EZH2 expression was detected in the hippocampus, showing significant differences between the two groups. Animal experiments were then conducted to confirm the hypothesis.

### Pretreatment with Sevoflurane Regulates Expression of EZH2 in Rat Hippocampus

We hypothesized that sevoflurane would improve PTSD symptoms by enhancing EZH2 expression in the hippocampus of PTSD rats. To confirm this, western blotting and immunofluorescence were used to detect the expression of EZH2 and determine the number of immunoreactive cells in each group (Fig. 2a, c). This showed the presence of greater numbers of immunoreactive cells in the control group than in the PTSD group. The PS group displayed more immunoreactive cells than the PTSD group, while the PSE group showed an obvious decrease in immunoreactive cells compared with the PSD group, indicating that administration of the inhibitor effectively reduced the expression of EZH2 (Fig. 2b). The western blot showed no difference in protein levels (Fig. 2d). The above experiments indicated that sevoflurane pretreatment mitigated the reduction in EZH2 levels in PTSD rats, and the use of EZH2 inhibitors eliminated the effect of sevoflurane.

### Upregulation of EZH2 Improves the Behavior of PTSD Rats

**Fig. 3:**
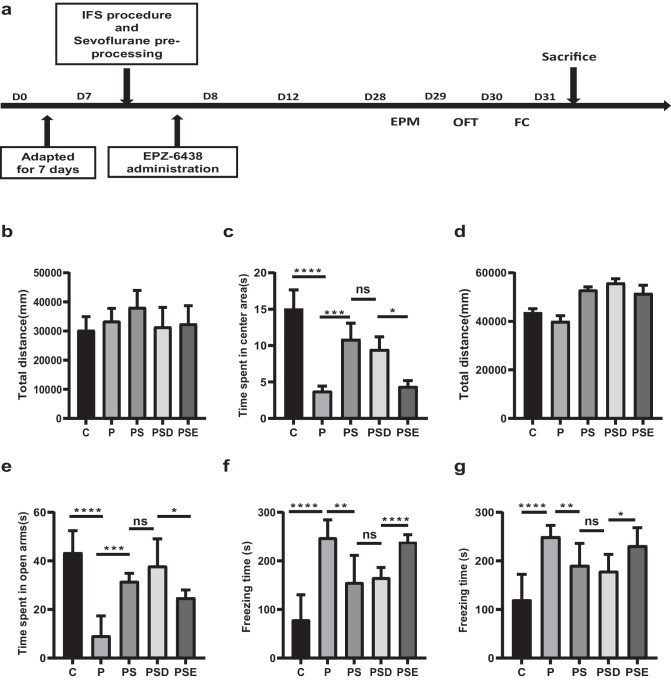
Upregulation of EZH2 improves PTSD-related behavior in PTSD rats. **a** Diagram showing the IFS procedure and administration of sevoflurane and EPZ-6438, and the timeline of the behavioral tests. Total distance moved and time spent in the central area in the OFT (**b**–**c**) and EPM (**d**–**e**) tests. **f**–**g** Freezing times of rats in each group in the contextual and conditional test. *p < 0.05, **p < 0.1, ***p < 0.01, ****p < 0.001; ns represents no significant difference. All the data are shown as means ± SEM. Data were analyzed via one-way analysis of variance

Administration of sevoflurane was used to ameliorate the symptoms of PTSD, and EPZ-6438 was used to reduce EZH2 expression (Fig. 3a). To further explore the effects of sevoflurane and EZH2, behavioral tests were performed to assess anxiety and fear memory in rats. These showed no significant differences in the total distances moved in the OFT and EPM tests, indicating no difference in the rats’ ability to move (Fig. 3b, d). Notably in these tests, compared to the control group, PTSD rats spent less time in the central area than rats in the control group (Fig. 3c, e). Fear conditioning tests showed the same results, indicating that the IFS procedure was effective in producing long-term anxiety and persistent fear memory (Fig. 3f, g).

In contrast, there was a marked improvement in the effects of PTSD behavioral symptoms after pretreatment with sevoflurane. The results showed that sevoflurane had a significant effect on PTSD. Notably, the administration of EPZ-6438 reversed the effect of sevoflurane to some extent. All these results show that sevoflurane can improve PTSD-related behavior in rats by modulating EZH2 expression.

### Sevoflurane Pretreatment Alleviates Apoptosis in Rat Hippocampus via Upregulation of EZH2

**Fig. 4:**
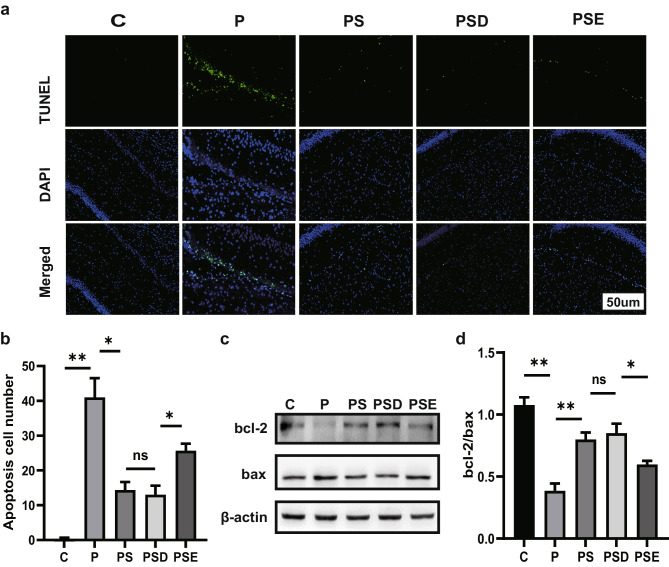
Sevoflurane pretreatment alleviates apoptosis in rat hippocampus via upregulation of EZH2. **a** TUNEL results for each group. Apoptotic cells are shown in the images. **b** Bar chart showing numbers of apoptotic cells in each group. **c** Western blotting of Bcl-2 and Bax in each group. **d** Bar chart of the Bcl-2/Bax ratio in each group. *p < 0.05, **p < 0.1, ***p < 0.01, ****p < 0.001; ns represents no significant difference. All data are shown as means ± SEM. Data were analyzed via one-way analysis of variance

In this study, we performed TUNEL and western blotting to verify the effects of sevoflurane. The PS group treated with sevoflurane showed fewer apoptotic cells in the TUNEL assay than the PTSD group (Fig. 4a, b). The Bcl-2/Bax ratio was higher in the PS group than in the PTSD group (Fig. 4c, d). Notably, after treatment with EPZ-6438, the PSE group showed greater numbers of apoptotic cells in the hippocampus, along with a lower Bcl-2/Bax ratio. All the results further demonstrated that pretreatment with sevoflurane influenced apoptosis in the rat hippocampus through regulation of EZH2 expression.

### Upregulation of EZH2 Alleviates Apoptosis via the AKT/mTOR Pathway

**Fig. 5:**
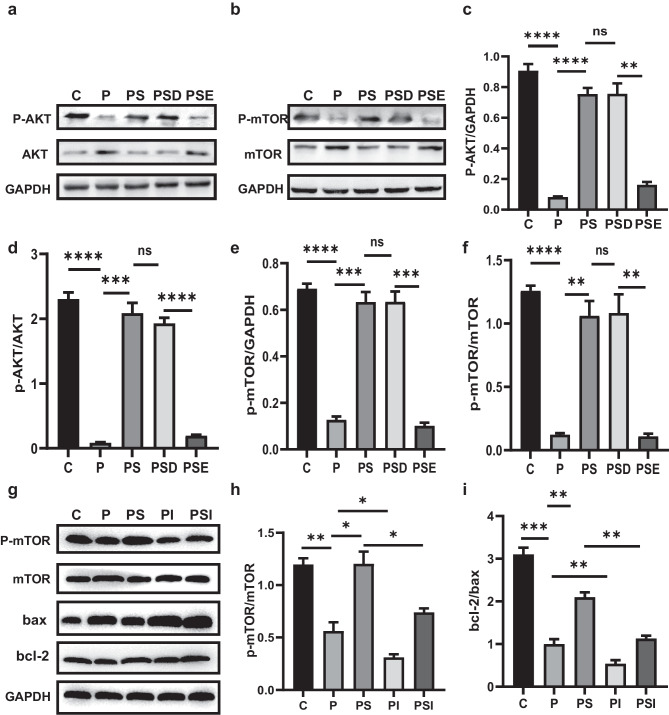
Upregulation of EZH2 alleviates apoptosis via the AKT/mTOR pathway. **a**–**b**, **g** Western blotting showing densities of p-AKT, AKT, p-mTOR, and mTOR in each group. **c**–**f**, **h**–**j** Bar graphs of p-AKT/GAPDH, p-AKT/AKT, p-mTOR/GAPDH, p-mTOR/mTOR, and Bcl-2/Bax ratios in each group. *p < 0.05, **p < 0.1, ***p < 0.01, ****p < 0.001; ns represents no significant difference. All data are shown as means ± SEM. Data were analyzed via one-way analysis of variance

EZH2 expression affects the process of memory reconsolidation by regulating the activation of the AKT/mTOR pathway, which may be an important epigenetic mechanism in PTSD. To further explore the mechanism, western blotting was used to assess the expression of proteins associated with the AKT/mTOR pathway (Fig. 5a–b). Phosphorylation was found to be enhanced after sevoflurane treatment. We then used EPZ-6438 to inhibit the expression of EZH2, resulting in a significant reversal of the increase in phosphorylation. The inhibition of mTOR activation was further verified by an intervention experiment (Fig. 5h), and the results showed that the activation level of mTOR significantly affected the survival state of rat brain (Fig. 5i). These results demonstrated that sevoflurane pretreatment alleviated apoptosis in the rat hippocampus by enhancing phosphorylation in the AKT/mTOR pathway, which was dependent on EZH2 expression.

### Pretreatment with Sevoflurane Can Effectively Enhance Synaptic Plasticity via Upregulation of EZH2

**Fig. 6:**
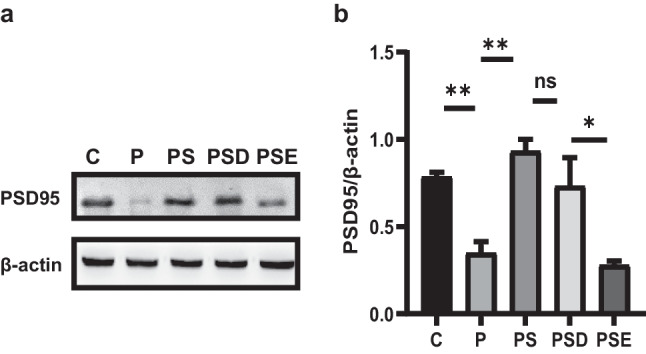
Pretreatment with sevoflurane enhances synaptic plasticity via upregulation of EZH2. **a** Western blotting showing PSD95 expression in each group. **b** Quantification of PSD95 staining densities in each group. *p < 0.05, **p < 0.1, ***p < 0.01, ****p < 0.001; ns represents no significant difference. All data are shown as means ± SEM. Data were analyzed via one-way analysis of variance

To demonstrate the effects of sevoflurane on synaptic plasticity, we assessed the expression of PSD95 in the hippocampi in each group by western blotting. The expression of PSD95 was significantly increased in the PSD group compared with the P group (Fig. 6a–b), implying that sevoflurane influenced synaptic plasticity. To further explore the role of EZH2 in synaptic plasticity, we measured PSD95 expression in the PSE group, observing that it was significantly increased compared with the PSD group. The results show that the administration of sevoflurane restored synaptic plasticity in the rat hippocampus via the upregulation of EZH2 expression.

## Discussion

Memory can be divided into short-term and long-term memory, with long-term memory in rodents lasting up to 4 weeks. Fear memories and anxiety associated with PTSD have been reported to persist for more than 21 days after a traumatic event (Ji et al. 2014). Given the limited treatment options for PTSD, it is essential to investigate novel approaches to reduce the prevalence of PTSD. In this study, we investigated the potential of sevoflurane pretreatment in reducing the symptoms of PTSD in rats. We found that pretreatment with sevoflurane significantly enhanced phosphorylation of the AKT–mTOR pathway by restoring the level of EZH2 expression, alleviating apoptosis in the rat hippocampus, and improving neurosynaptic plasticity. As a result, the symptoms of PTSD were significantly ameliorated. These findings provide a promising new strategy for treating PTSD and could potentially be applied to humans in the future.

Sevoflurane is a volatile anesthetic that blocks sodium and calcium channels. In cerebral ischemia–reperfusion rats, sevoflurane was observed to inhibit the processes of autophagy and apoptosis, and improve both behavior and awareness (Shi et al. 2020). In clinical studies, sevoflurane pretreatment has been demonstrated to have cardioprotective effects in coronary artery surgery (Cromheecke et al. 2006). However, sevoflurane has also been shown to have neurotoxic effects in neonatal mice and to impair the working memory of aged rats by reducing the functional connections of excitatory neurons in the prefrontal cortex (Xu et al. 2018). Most notably, sevoflurane is widely used in pediatric anesthesia because of its pleasant smell. Sevoflurane has been reported to cause a high incidence (up to 80%) of emergent delirium in children (Moore and Anghelescu 2017), which may result from an immature mental and neural status, drug residues, and the rapid and excessive release of catecholamines. However, a recent international multicenter clinical trial showed no significant effect of sevoflurane on neurodevelopment five years after exposure in infancy (McCann et al. 2019). In summary, the concentration of sevoflurane, the duration and times of exposure, the developmental stages of the brain, and the status of the nervous system can all affect its efficiency. In accordance with a previously described protocol, we chose adult rats and adopted a suitable concentration and duration of sevoflurane administration in this study. Significant improvements were observed in the behavior of PTSD rats after treatment with sevoflurane.

EZH2 can alter the expression of genes and further regulate epigenetic events via the regulation of trimethylated histone H3 at lysine 27 (H3K27me3), as well as activate downstream genes independently of PCR2. In vivo experiments revealed that reduced EZH2 expression in neural stem cells and progenitor cells causes a persistent decrease in neuronal production via the PTEN-AKT-mTOR signaling pathway. EZH2-null mice showed obvious impairments in spatial learning and memory, which provided important insights into the function of EZH2 in adult neurogenesis. EZH2 was also shown to participate in complex cognition and the cognitive process of actively forgetting prior memories. These results suggest that regulation of the expression and activity of EZH2 may help in treating neurological disorders (Zhang et al. 2014). In contrast, however, EZH2 overexpression was found to promote microglial activation and intensify cognitive dysfunction (Chen et al. 2019; Wang et al. 2020). Inhibiting the overexpression of EZH2 can also alleviate inflammation and glycolysis in acute spinal cord injury (Ni et al. 2021). In our study, preconditioning of rats with sevoflurane significantly counteracted reduced EZH2 expression in PTSD rats. We further showed that this modulation of EZH2 expression was followed by increased phosphorylation of the AKT–mTOR signaling pathway. Notably, inhibition of EZH2 tended to offset the effects of sevoflurane in PTSD rats, which provided further information on the mechanism of EZH2 expression following sevoflurane administration.

Stress and stimuli can induce apoptosis, a programmed cell death that may be associated with the neurochemical changes triggered by stress-related disorders (Engelbrecht et al. 2010). Intense stress induces the release of glutamate, and the imbalance between excitatory and inhibitory transmitters (mainly glutamate and GABA) may promote the apoptosis of hippocampal neurons, which plays an important role in the pathogenesis of PTSD (Gao et al. 2014). In our study, sevoflurane treatment reduced apoptosis in the hippocampus by restoring the expression of EZH2. Notably, EZH2 induced the activation of the AKT-mTOR signaling pathway. A previous study reported that inhibition of mTOR signaling resulted in increased cell death due to apoptosis and autophagy (Degtyarev et al. 2008). Epigenetic modification (in the form of histone methylation) was demonstrated to regulate the expression of mTOR during learning (dependent on synaptic plasticity) in neurons (Jarome et al. 2018). Additionally, the PI3K/AKT pathway was found to regulate mTOR activation and thus influence both autophagy and apoptosis (Yano et al. 2001). It is important to note that the excessive formation of autophagosomes can also increase apoptosis in cells (Ma et al. 2011). In our study, we also explored the effect of sevoflurane on synaptic plasticity. The decline in neurotrophic and synaptic proteins may lead to significant deficits in the retrieval of cue and contextual fear memories. The results demonstrated significant improvements in synaptic plasticity in PTSD rats after sevoflurane pretreatment and consequent EZH2 regulation.

It is important to note that we did not include a sevoflurane intervention control group in this study. The primary aim was to investigate the potential benefits of sevoflurane intervention on PTSD and to determine its underlying mechanisms. In addition, previous studies have confirmed that sevoflurane has no significant effects on control samples (Wang et al. 2021). Pretreatment with sevoflurane has been shown to effectively reduce the incidence of PTSD. The results suggest the potential of the drug for the prevention of PTSD in individuals at risk of predictable trauma. Sevoflurane could also be applied in surgery to reduce postoperative stress disorders caused by surgery. Therefore, further research is needed to fully understand the effects of sevoflurane.

## Supplementary Information

Below is the link to the electronic supplementary material.

Supplementary file1 (JPG 343 KB)

## Data Availability

Some or all data, models, or code generated or used during the study are available from the corresponding author by request.
