# Supplementary figures and images for: Sevoflurane Preconditioning Alleviates Posttraumatic Stress Disorder—Induced Apoptosis in the Hippocampus via the EZH2-Regulated Akt/mTOR Axis and Improves Synaptic Plasticity
Source: J Mol Neurosci. 2023 Mar 17;73(4-5):225–36. doi: 10.1007/s12031-023-02114-1 (PMC10200787; doi:10.1007/s12031-023-02114-1)

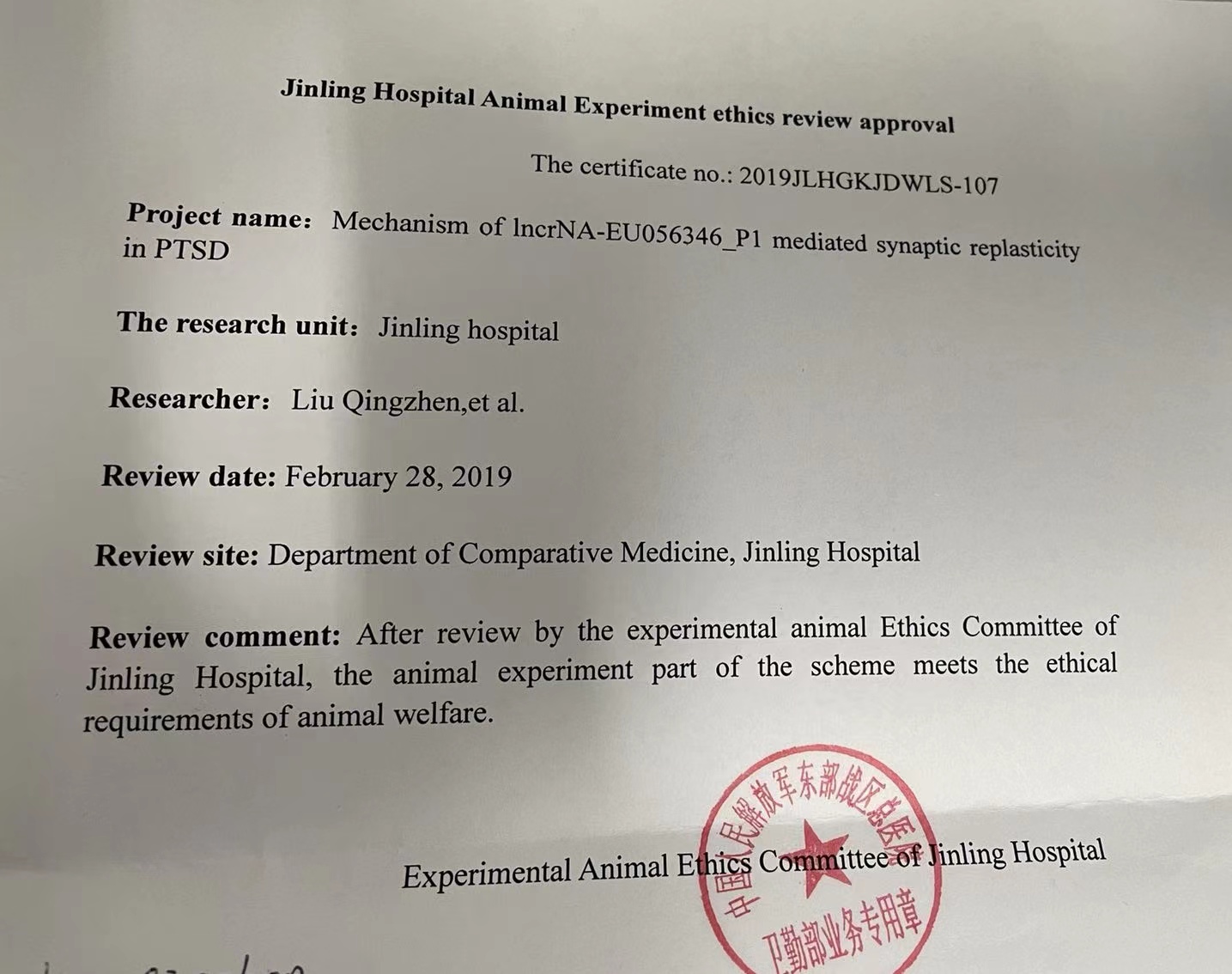

Supplement: Supplementary file 1 — Supplementary file1 (JPG 343 KB) [file 12031_2023_2114_MOESM1_ESM.jpg]
